# NLRP3 Inflammasome in Diabetic Cardiomyopathy and Exercise Intervention

**DOI:** 10.3390/ijms222413228

**Published:** 2021-12-08

**Authors:** Yi Sun, Shuzhe Ding

**Affiliations:** 1 Key Laboratory of Adolescent Health Assessment and Exercise Intervention of Ministry of Education, East China Normal University, Shanghai 200241, China; ysun@tyxx.ecnu.edu.cn; 2 College of Physical Education and Health, East China Normal University, Shanghai 200241, China

**Keywords:** NLRP3 inflammasome, DCM, exercise, P2X7, inflammation

## Abstract

Diabetic cardiomyopathy (DCM), as a common complication of diabetes, is characterized by chronic low-grade inflammation. The NLRP3 inflammasome is a key sensor mediating innate immune and inflammatory responses. However, the mechanisms initiating and promoting NLRP3 inflammasome activation in DCM is largely unexplored. The aim of the present review is to describe the link between NLRP3 inflammasome and DCM, and to provide evidence highlighting the importance of exercise training in DCM intervention. Collectively, this evidence suggests that DCM is an inflammatory disease aggravated by NLRP3 inflammasome-mediated release of IL-1β and IL-18. In addition, chronic exercise intervention is an effective preventive and therapeutic method to alleviate DCM via modulating the NLRP3 inflammasome.

In 2017, about 451 million adults lived with diabetes worldwide, and this number was predicted to increase to 693 million by 2045 [1]. In addition, it is estimated that almost half of people living with hyperglycemia are undiagnosed. Diabetic cardiomyopathy (DCM) is one of the main causes of death among the various complications of diabetes. The molecular mechanisms of DCM include hyperglycemia, insulin resistance, fatty acids, oxidative stress, mitochondrial dysfunction, endothelial dysfunction, etc. [2]. Furthermore, it is believed that inflammation is already present in the early phase of diabetes and is a key promoting factor of DCM [3].

Recent evidence showed that the NLRP3 inflammasome was highly expressed in cardiac cells. However, the factors initiating NLRP3 inflammasome activation remain elusive. In addition, increasing evidence has revealed that the signaling pathway of the NLRP3 complex is influenced by different regimens of exercise [4]. DCM is also known to be alleviated by chronic exercise training [5,6]. This evidence together points to the fact that the NLRP3 inflammasome is a promising molecular complex mediating the protective effect of exercise in DCM.

## 1. NLRP3 Inflammasome Biology and Pyroptosis

Activation of the innate immune system starts with recognition of pathogen-associated molecular patterns (PAMPs) and damage/danger-associated molecular patterns (DAMPs) [7]. In response to pathogens or stressful stimuli, pattern recognition receptors (PRRs) that are expressed on macrophages, monocytes, neutrophils and epithelial cells are activated [8]. Nod-like receptors (NLRs) are one family of PRRs that are involved in identifying PAMPs and DAMPs [9]. NLRs are divided into four subfamilies. The NLRP subfamily is mainly involved in the development of the inflammasome complex, and NLRP3 is the most characteristic one.

The NLRP3 inflammasome consists of NLRP3, apoptosis-associated speck-like protein containing a CARD domain (ASC) and pro-caspase-1 [10]. NLRP3 is the receptor protein, serving as a sensor of various activators [7]. ASC is the adaptor protein, working as a bridge between NLRP3 and pro-caspase 1. The ASC protein is actually not considered to have inflammatory activities outside the NLRP3 inflammasome [11]. The activation of the NLRP3 inflammasome includes two steps [12]. In the first step, upon priming signal (microbial or endogenous molecules such as LPS or oxLDL), production of NLRP3, pro-IL-1β and pro-IL-18 are enhanced by NF-κB transcription. In the second step, several substances such as ATP, toxins, mitochondrial DNA, and uric acid crystals work as activating signals. Upon activation, NLRP3 is oligomerized and forms an interaction with ASC. ASC then interacts with pro-caspase-1, triggering autocleavage of pro-caspase-1 to become active caspase-1 and ultimately leading to cleavage and maturation of pro-IL-β and pro-IL-18 to become IL-β and IL-18 [13].

Besides involvement in cytokine maturation, the NLRP3 inflammasome also plays a central role in a novel form of cell death, called pyroptosis. Pyroptosis is a newly discovered form of programmed necrosis, and is characterized by cellular lysis and release of proinflammatory cytokines including IL-1β and IL-18 [14]. The two proposed pathways of pyroptosis include the canonical pathway and noncanonical pathway [15]. In the canonical pathway, caspase-1 is activated, leading to cleavage of pyroptosis executioner gasdermin D (GSDMD). The N-terminal (p30 fragment) of GSDMD is the active part, exhibiting membrane pore-forming activity by binding to phosphoinositides in the plasma membrane [16]. Cell swelling and lysis occur next, causing release of IL-1β and IL-18. In the noncanonical pathway, lipopolysaccharide (LPS) is delivered to the cytosol to activate mouse caspase-11 [17]. Caspase-11 then interacts with LPS, cleaving GSDMD to generate active GSDMD-N. Besides mouse caspase-11, human caspase-4 and -5 also contribute to the noncanonical pathway of pyroptosis by binding to LPS [18]. Meanwhile, caspase-11 also triggers NLRP3 inflammasome activation as well as caspase-1-dependent release of IL-1β and IL-18.

## 2. The NLRP3 Inflammasome in the Development of DCM

Glucose has been shown to be a potent activator of the NLRP3 inflammasome [10]. In addition, as proinflammatory cytokines, IL-1β and IL-18 are actively involved in the initiation and progression of diabetes and diabetic complications [3].

### 2.1. DCM, a Severe Complication of Diabetes

Diabetes mellitus (diabetes, DM) is a group of metabolic disorders characterized by elevated blood glucose levels [1]. Diabetic patients suffer from a series of life-threatening complications affecting the heart, eyes, kidneys and nerves. Heart failure is the most common cardiovascular complication of diabetes and is the major cause of mortality for diabetic patients [19]. However, heart failure in diabetic patients could not be solely explained by increased incidence of atherosclerosis, hypertension or coronary heart disease. Therefore, Lundbaek introduced a new disease in 1954, named diabetic cardiomyopathy [20].

DCM is defined as abnormalities of myocardial structure and function in diabetic patients that are not solely attributed to hypertension, congenital heart diseases or coronary artery diseases [2,21]. DCM patients usually exhibit left ventricular hypertrophy, myocardial cell death and myocardial fibrosis. It is generally accepted that diastolic dysfunction happens in the early stage of DCM. Diastolic dysfunction is considered a predictor of bad prognosis in heart failure with reduced ejection fraction [22,23]. Combined systolic and diastolic dysfunction start to show up in the second stage of DCM [19]. The molecular mechanisms accounting for DCM include hyperglycemia, hyperlipidemia, insulin resistance, oxidative stress, low-grade inflammation, mitochondrial dysfunction, endoplasmic reticulum stress (ERS) and endothelial dysfunction [24].

### 2.2. The NLRP3 Inflammasome and Diabetes

The innate immune system is the initial barrier to protect the body from infection and injury. The inflammatory response is aimed to clear stressors and restore tissue homeostasis [25]. However, prolonged and overwhelmed activation of the immune system by nutrient overload promotes metabolic diseases. Numerous studies have shown that the NLRP3 inflammasome is involved in the pathogenesis and progression of both type 1 diabetes (T1DM) and type 2 diabetes (T2DM). Upon activation of NLRP3, a large amount of proinflammatory cytokines including IL-1β and IL-18 are secreted, aggravating glucose intolerance and insulin resistance [10]. Studies have shown that the mRNA expressions of NLRP3, ASC and pro-IL-1β as well as protein levels of NLRP3 and IL-1β were all increased in the pancreatic lymph nodes of T1DM mice [26]. The fact that IL-1R^−/−^ mice were protected more from developing T1DM upon streptozotocin (STZ) treatment also suggested that NLRP3-IL-1β signaling contributed to pancreatic islet inflammation, β cell damage, and ultimately T1DM development [26]. With regard to T2DM, increasing evidence has indicated the association between chronic low-grade inflammation and T2DM, especially highlighting the essential role that the NLRP3 inflammasome plays in the development of obesity, insulin resistance and T2DM. The fact that NLRP3^−/−^ and Pycard^−/−^ mice both had lower blood glucose and insulin levels suggested that absence of the NLRP3 inflammasome was linked to improved glucose homeostasis [27]. A study with *db*/*db* mice and the NLRP3 selective inhibitor MCC950 also indicated that NLRP3 and pro-inflammatory cytokines contributed to vascular dysfunction in T2DM [28]. A detailed review on the role of the NLRP3 inflammasome in diabetes can be found in Ding et al. [10].

### 2.3. NLRP3 Inflammasome and DCM

Numerous studies have shown that the progression of DCM is associated with chronic inflammation and cardiac cell death, and might ultimately lead to heart failure [29]. Cardiac inflammation is an early and notable response in diabetes and is involved in the development of DCM. The progression of DCM has been linked to the NLRP3 inflammasome [3]. The mRNA expressions of NLRP3, ASC, caspase-1 and IL-1β were all found to be higher in the hearts of diabetic mice [30].

**Figure 1:**
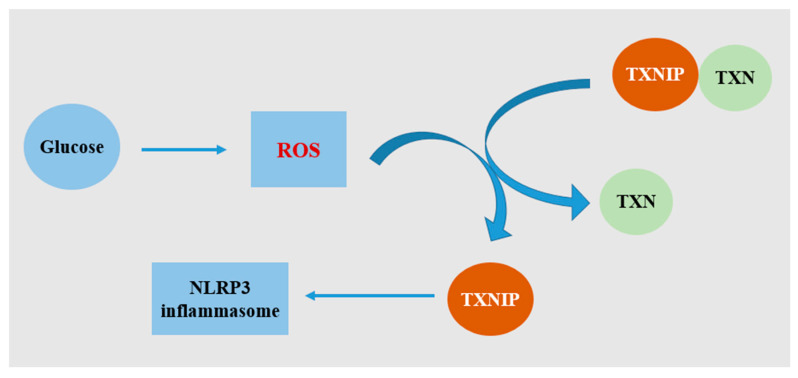
Schematic diagram of activation of the NLRP3 inflammasome by TXNIP under hyperglycemia. Glucose stimulates excessive ROS generation. TXNIP usually binds and negatively regulates the activity of TXN (thioredoxin). Under stress, TXNIP is dissociated from TXN. TXNIP then binds NLRP3 directly, leading to assembly of the NLRP3 inflammasome.

NLRP3 gene silencing alleviated cardiac inflammation and fibrosis, in addition to improving cardiac function in diabetic rats [31]. To be more specific, the protein levels of cardiac NLRP3, active caspase-1, and IL-1β were reduced in the NLRP3-miRNA group. An in vitro study on H9c2 cells showed that the increase of NLRP3, ASC, caspase-1 and IL-1β mRNA expressions followed a glucose concentration-dependent pattern, confirming that glucose was a potent activator of the NLRP3 inflammasome [3,31]. A further study showed that NF-κB and thioredoxin-interacting protein (TXNIP) mediated NLRP3 inflammasome activation, which was caused by high glucose-induced ROS generation (Figure 1) [31]. Cardiomyocytes are terminally differentiated cells. Therefore, death of cardiomyocytes is a deadly molecular event in the progression of DCM, leading to loss of contractile units [6]. As a type of inflammation-mediated cell death, pyroptosis is considered to precede cardiac remodeling and dysfunction, and is induced by mitochondrial damage and cardiac lipotoxicity [6]. Inhibiting NLRP3 expression in H9c2 cells caused a decrease in protein expressions of caspase-1 and IL-1β under high glucose, as well as lower cell death rate [31].

Because of the essential role of the NLRP3 inflammasome in DCM progression, studies have been performed to examine the effect of drug administration on DCM via regulating the NLRP3 inflammasome. For example, TXNIP expression, NLRP3 inflammasome activation, IL-1β and IL-18 were all found to be suppressed in DCM mice after twelve-week administration of the anti-aging protein Klotho. At the same time, cardiac fibrosis, apoptosis and dysfunction were all improved [32]. Similarly, rosuvastatin alleviated enhanced expressions of TXNIP, NLRP3, ASC and IL-1β in left ventricular tissue of diabetic rats [33]. However, the cardioprotective effect of rosuvastatin was abrogated with NLRP3-miRNA treatment, confirming that rosuvastatin alleviated cardiac dysfunction in DCM rats via suppressing NLRP3 inflammasome [33]. Besides the NLRP3 inflammasome, other inflammasomes are also involved in the inflammatory pathway of DCM as well as in cardiomyocyte pyroptosis [2]. AIM2 is a member of the HIN200 protein family. AIM2 could form a platform with ASC, activate caspase-1, and cause the maturation of IL-1β and IL-18. Wang et al. found that AIM2, ASC, caspase-1_p10 + p12_, IL-1β_p17_ and GSDMD-N were all elevated in the hearts of diabetic rats, and the above-mentioned parameters were all reduced upon inhibition of AIM2 [34]. Therefore, like the NLRP3 inflammasome, AIM2 also plays an important role in DCM via the GSDMD pathway.

### 2.4. The NLRP3 Inflammasome as the Link between Diabetes, DCM and Heart Failure

As a leading cause of death, heart failure has been shown to be related to chronic systemic and cardiac inflammation. Inflammation is considered to be the cause and driver of alterations in the extracellular matrix, cardiac fibrosis, and contractile dysfunction [35]. Circulating levels of pro-inflammatory cytokines such as TNF-α were shown also to correlate with disease severity [36]. However, most studies attempting to treat heart failure directed at reducing inflammation ended up with failure, possibly due to the complex immune and inflammatory responses during the late stages of heart failure [35]. Therefore, it is essential that inflammation-related heart conditions should be seriously treated before symptoms of heart failure show up. Evidence has shown that diabetic patients have more than twice the risk of developing heart failure than patients without diabetes [37]. In addition, cardiac inflammation has been implicated in the pathophysiology of DCM too. Therefore, considering the essential role that the NLRP3 inflammasome plays in DCM [3], targeting the NLRP3 inflammasome pathway might be a plausible treatment to reduce the risk of developing heart failure in diabetes [10,37]. Indeed, IL-1β is a key pro-inflammatory mediator of β-cell damage in T2DM [38]. A recent large-scale clinical trial showed that the IL-1β inhibitor canakinumab caused a dose-dependent reduction in the risk of hospitalization for heart failure [39]. Similarly, dapansutrile, as the inhibitor of the NLRP3 inflammasome was shown to be effective in improving left ventricular EF and exercise time after 14 days of treatment in patients with heart failure [40].

### 2.5. The P2X7 Receptor, NLRP3 Inflammasome and DCM

It has long been noticed that phosphorylated compounds play a role in inflammation and immunity via stimulating intracellular second messengers such as Ca^2+^. A later investigation showed that the P2X7 receptor (P2X7R) might be a mediator in extracellular ATP-induced inflammation [41]. The P2X7 receptor, originally known as the P2Z receptor, is a member of the purinergic receptor families. P2X7R has a low affinity for ATP. Therefore, the activation of the P2X7 receptor requires a high level of ATP, usually up to millimolar range [41,42]. Ligation of P2X7R to ATP causes channel opening, influx of Ca^2+^/Na^+^ and efflux of K^+^ [43]. As the most well-studied purinergic receptor, P2X7R is also involved in the activation of the NLRP3 inflammasome [41]. Following cellular stress or tissue injury, ATP, as a DAMP, is released by cells. ATP was shown to be a strong stimulus for IL-1β release from macrophages in mice [44]. Further study showed that activation of P2X7R caused K^+^ efflux, which induced NLRP3 inflammasome assembly and the subsequent maturation and secretion of IL-1β and IL-18 [41,45]. Confocal microscopy and immunoprecipitation assays confirmed that P2X7R and NLRP3 physically interacted with each other [46]. Mice deficient in P2X7R showed compromised IL-1β production in response to LPS [47]. P2X7R also induced ROS production through activation of NAPDH oxidase [48]. Fontanils et al. also pointed out that influx of Ca^2+^ could explain the P2X7R-dependent ROS generation [49]. Moreover, the P2X7R antagonists caused inhibition of ATP-induced cation uptake by 75–100%, as well as a complete inhibition of ATP-induced ROS production [50].

There are two types of ATP release mechanisms, via exocytosis or through ion channels on plasma membrane [51]. During the resting state, the low amounts of extracellular ATP are rapidly degraded by ecto-ATPases. However, under hypoxia, mechanical stress or cell death, a large amount of ATP is released through pannexin-1, activating P2X7. The ligation of ATP and P2X7R in turn triggers pannexin-1 to come into a large, ATP-permeable conformation [52]. Because of the low affinity of P2X7R to ATP, pannexin-1 and P2X7R also physically interact with each other, revealed by proximity ligation assay [53].

**Figure 2:**
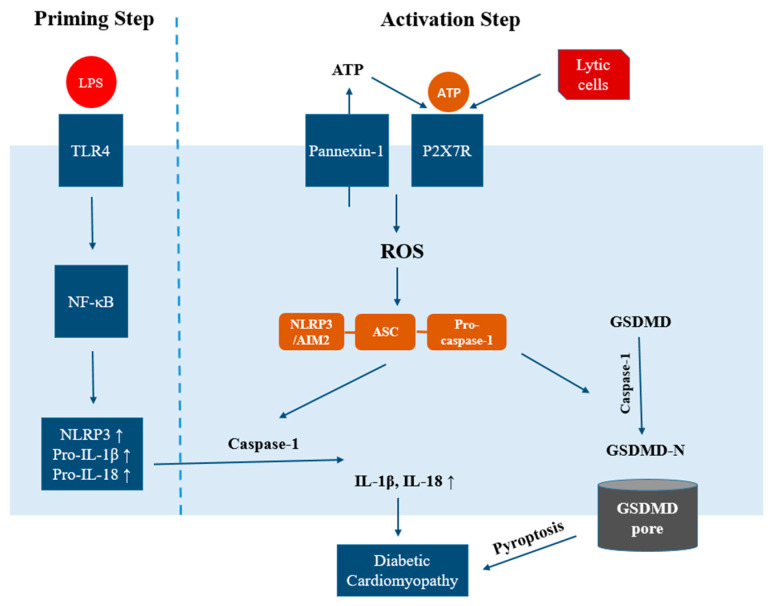
Schematic summary of the involvements of P2X7R, NLRP3 inflammasome and pyroptosis in DCM. Two steps are required for the processing and release of the mature form of IL-1β and IL-18. In the first (priming) step, recognition of inflammatory stimuli by the membrane receptor TLR4 triggers the activation of NF-κB, which then induces transcriptions of NLRP3, pro-IL-1β and pro-IL-18. In the second (activation) step, an activation signal triggers assembly of the NLRP3 inflammasome. Caspase-1 then mediates the maturation and secretion of IL-1β and IL-18. In addition, caspase-1 cleaves GSDMD to release GSDMD-N, which generates membrane pores. The release of IL-1β and IL-18, as well as pyroptosis, together contribute to the pathogenesis of DCM. In DCM, the NLRP3 inflammasome is possibly activated via P2X7R. A high level of ATP released from Pannexin-1 channels activates P2X7R, generating excessive ROS and promoting NLRP3 assembly.

The P2X7R-NLRP3 axis (Figure 2) actively participates in the pathogenesis of many diseases, including diabetes [54], atherosclerosis [55], glomerulonephritis [41] and emphysema [56]. In white adipocytes, ATP release via pannexin-1 is stimulated by high extracellular glucose and is inhibited by insulin [51]. Tozzi et al. proposed that during T2DM when insulin action was compromised, an abnormally high amount of ATP was released from white adipocytes, affecting cell functions [51]. Similarly, pannexin-1 expression and enhanced ATP release were also observed following myocardial ischemia-reperfusion [57]. An in vitro study utilizing H9c2 cells suggested that inhibition of P2X7R significantly decreased the expressions of NLRP3, caspase-1, and IL-1β secretion [58]. The P2X7R inhibitor also improved cardiac fibrosis and apoptosis induced by palmitic acid. In addition, Zhang et al. showed that high-glucose medium caused collagen synthesis via activation of ROS and P2X7R in rat cardiac fibroblasts [59]. Collectively, P2X7R could be a potential target for alleviating cardiac fibrosis in DCM via modulating the NLRP3 inflammasome.

## 3. Exercise Intervention for Diabetic Cardiomyopathy

If hyperglycemia is not corrected, diabetic patients might end up with cardiac hypertrophy and myocardial dysfunction [60]. Physical activity is a basic treatment recommended for diabetic patients [61]. A meta-analysis based on twelve aerobic training studies and two resistance training studies revealed that exercise training could effectively lower the HbA1c level, even when body mass was not significantly altered [62]. Moreover, exercise training of more than 150 min per week was associated with greater HbA1c decline [63].

### 3.1. Exercise Intervention to Regulate the NLRP3 Inflammasome

It is well known that regular physical activity exerts many health benefits, partly through regulating inflammation. The NLRP3 inflammasome plays an important role in innate immunity by responding to various microbial and endogenous products, and its structure and function is also tightly modulated by exercise intervention (Table 1).

The NLRP3 inflammasome seems to be activated during the early stage of acute exercise, as well as during the recovery period. The expressions of NLRP3 and IL-1β in mice myocardium were increased following 45 min acute exercise, and at 12 h and 24 h post exercise [64]. However, NLRP3 and IL-1β were not different from the resting level following 90 min or 120 min exercise, or at 36 h or 48 h post exercise. It is generally accepted that mitochondrial ROS is a potent activator of the NLRP3 inflammasome. However, the fact that upregulations of NLRP3 and IL-1β take place early during acute exercise, and that a surge of ROS generation comes next suggest that ROS and NLRP3 might form a vicious cycle of inflammatory response [64,65]. The expression of NLRP3 following exercise also depends on the exercise regimen and intensity. The effects of acute and chronic exercise of different intensities on NLRP3 expression in young men have been compared. It turned out that acute aerobic exercise of high intensity increased NLRP3 mRNA expression and serum IL-1β and IL-18, while acute exercise of moderate intensity did not change the above parameters. When it comes to chronic exercise, decreased and increased expressions of NLRP3 gene, serum IL-1β and IL-18 were observed with moderate and high intensity exercise, respectively [4]. Similarly, even though acute exercise did not affect IL-18 mRNA expression in the adipose tissue at 0 h, 2 h or 10 h post-exercise in non-obese subjects, eight-week exercise training caused reduced IL-18 mRNA content in the adipose tissue in obese subjects [66]. Compared to studies on NLRP3, there have been very few studies regarding the ASC gene response to exercise intervention. In one study, the combined effect of hypocaloric diet and moderate exercise training on inflammation level in the obese individuals was investigated. The study showed that a diet-exercise intervention decreased ASC mRNA expression [67]. In addition, a negative correlation was observed between the delta of ASC mRNA expression and IL-10 levels. Therefore, it was proposed that the ASC gene might be a molecular marker in response to exercise intervention for the obese individuals. The expression of the NLRP3 inflammasome is also regulated by resistance training. For elderly people, eight-week resistance training reduced NLRP3 expression and the caspase-1/procaspase-1 ratio in peripheral blood mononuclear cells (PBMCs), suggesting that NLRP3 inflammasome activation was prevented by resistance training [68]. The effects of aerobic vs. resistance training on inflammasome activation in mice were also compared. It turned out that resistance training attenuated the increased NLRP3 expression in adipose tissue and IL-18 serum level, which were induced by HFD [69]. In comparison to resistance training, aerobic training was more effective in lowering TNF-α, IL-1β and IL-18 level in the adipose tissue. Therefore, it seems that endurance training was more effective in suppressing macrophage and lymphocyte activation.

**Table 1:** The effects of different types of exercise intervention on NLRP3 inflammasome pathway.

Subjects	Model	Exercise Regimen	Main Findings	Ref
Rats	SD, male	Acute treadmill running, 45, 90 or 120 min	Myocardium NLRP3↑, IL-1β↑ following 45 min exercise and during recovery	[64]
Human	Healthy, young, male	(1) Acute moderate intensity: 50% HRmax for 30 min + 70% HRmax for 40 min, Nordic walking; (2) Acute high intensity, 70% HRmax for 30 min + 90% HRmax for 40 min, Nordic walking.	(1) No change in PBMCs NLRP3 mRNA, or serum IL-1β and IL-18; (2) PBMCs NLRP3 mRNA↑, serum IL-1β and IL-18↑.	[4]
Human	Non-obese, female/male	(1) 2-h exercise, 60% VO_2_max, cycling (2) 1.5-h exercise, 70% VO_2_max, cycling	No change in adipose IL-18 mRNA following exercise, or 2 h/10 h during recovery	[66]
Mice	C57BL/6, male, HFD	80% VO_2_max, treadmill running, 30 min/day, 5 times/week, 10 weeks.	Adipose mRNA of IL-1β↓, TNF-α↓, IL-18↓	[69]
Human	Healthy, young, male	(1) Moderate intensity: 50% HRmax for 30 min + 70% HRmax for 40 min, Nordic walking, 3 days/week, 3 months; (2) High intensity, 70% HRmax for 30 min + 90% HRmax for 40 min, Nordic walking, 3 days/week, 3 months.	(1) PBMCs NLRP3 mRNA↓, serum IL-1β and IL-18↓; (2) PBMCs NLRP3 mRNA↑, serum IL-1β and IL-18↑.	[4]
Human	Obese, male and female	High-intensity, 70% VO_2_max, rowing, 30 min/day, 3 days/week, 8 weeks.	Adipose IL-18 mRNA↓ after training. No change in plasma IL-18 after training.	[66]
Human	Obese, male and female	Hypocaloric diet & moderate-intensity, 65–75% HR, aerobic and resistance, 3–5 days/week, 16 weeks.	peripheral blood ASC mRNA↓, MCP-1↓, MIP-1β↓	[67]
Human	Elderly, male and female	Resistance exercise (leg press, biceps curl, pec deck), 60–80% 1RM, 2 sessions/week, 8 weeks.	PBMCs NLRP3↓, caspase-1/pro-caspase-1↓	[68]
Mice	C57BL/6, male, HFD	Isometric strength training, 3 min, 3 series with 1 min break, 5 times/week	Adipose NLRP3↓, serum IL-18↓	[69]

### 3.2. Exercise Intervention to Alleviate DCM

Exercise is established as an effective approach for management of diabetes and DCM [70]. The American Diabetes Association (ADA) and cardiovascular rehabilitation experts from 11 European countries recommend that diabetic patients perform aerobic and resistance exercise regularly, with a total of more than 150 min/week of moderate intensity exercise spread over 3–5 days per week [71,72]. The anti-inflammatory effect of exercise training is partly achieved by decreasing circulating inflammatory mediators. Twelve-week combined aerobic and resistance exercise was shown to lower insulin resistance index, expressions of TLR4, NF-κB _p65_ in monocytes, and serum IL-18 level in diabetic patients [73].

According to a cohort study, low cardiorespiratory fitness and physical inactivity were independent predictors of mortality in T2DM male patients [61]. Similarly, higher physical activity level was associated with reduced risk of coronary heart disease and ischemic stroke in diabetic women [74]. Interestingly, even faster walking pace by itself was associated with lower cardiovascular risks [74]. In fact, exercise is effective in reducing cardiac mortality in diabetic patients and increasing cardiac output and contractibility [75]. Exercise also normalized diastolic function in HFD-induced obese and T2DM mice [76,77]. Like DCM patients, obese subjects also showed diastolic dysfunction, which could be reversed by eight-week low intensity aerobic training (walking/cycling) [78]. For elderly heart failure patients with reduced ejection fraction, four-week endurance training was also highly effective in improving left ventricular diastolic function [23]. Long-term endurance training was generally considered effective in improving cardiac function in DCM patients. In one study, five out of eleven subjects recovered with normalized left ventricular diastolic function after twelve-month cycling training at 60–70% VO_2_max [79]. In another study, one-year gym- and home-based exercise intervention failed to improve the myocardial function of DCM patients [5]. However, the subjects that spent more time in moderate and vigorous activity did show improvement in myocardial tissue velocity and strain rate. Therefore, it seems that a greater exercise load higher than recommended by the current guideline is required to rescue myocardial function for DCM patients.

### 3.3. Exercise Intervention for DCM through the NLRP3 Inflammasome

**Figure 3:**
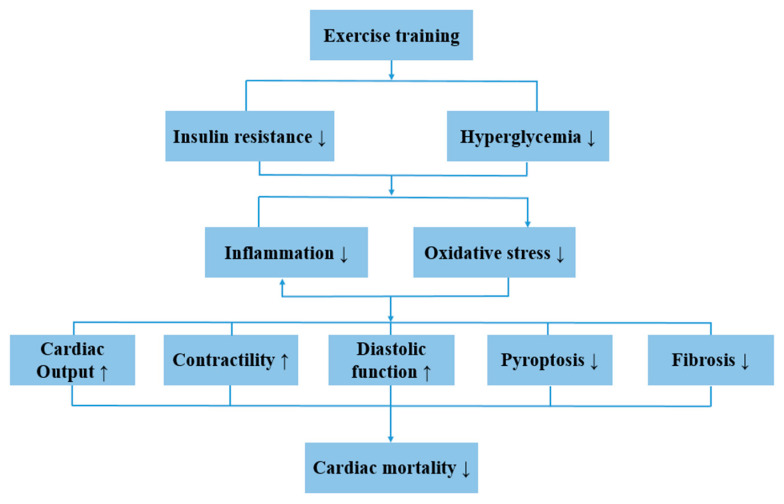
Schematic summary of the effect of exercise training on DCM. Exercise training causes alleviation of hyperglycemia and insulin resistance, which result in improvements of system and cardiac inflammation as well as reduced oxidative stress level. Therefore, cardiac output, cardiac contractility and diastolic function are improved, and pyroptosis and cardiac fibrosis are rescued.

It has long been noticed that supervised exercise training can improve T2DM by alleviating the expression of cytokines, such as resistin, IL-6 and IL-18 [80]. Recent studies indicated that the NLRP3 inflammasome was actively involved in exercise-mediated alleviation of DCM (Figure 3).

Obesity is a key contributor to the development of cardiovascular diseases and DCM, and chronic low-grade inflammation is considered a hallmark of obesity. Lee et al. found that expressions of NLRP3, caspase-1_p20_, caspase-1_p20_/caspase-1 and IL-1β were increased in the myocardium of HFD-induced obese mice, and were significantly inhibited by 12–14 weeks of voluntary running [81]. In an animal study, HFD for 20 weeks was sufficient to induce the DCM phenotype in mice, shown by impaired diastolic function. At the same time, the pro-fibrotic molecules TGFβ (transforming growth factor β-1) and β-MHC (β-myosin heavy chain) were also found to be elevated with HFD [6]. Evidence showed that NLRP3 inflammasome formation and activation in the left ventricles of mice was induced by HFD, revealed by increased expressions of NLRP3, ASC, pro-caspase-1, and IL-1β, and the above parameters were all inhibited by treadmill exercise [6]. Therefore, treadmill training was effective in preventing the development of DCM and alleviating cardiac pyroptosis. However, in the same study, treadmill training did not improve structural remodeling pathways, suggesting that correction of cardiac inflammation preceded cardiac structural alterations. The effect of exercise preconditioning on cardiac function in normal rats submitted to exhaustive exercise has also been investigated [82]. Compared to the exhaustive exercise group, expressions of TXNIP, NF-κB_p65_ and caspase-1 were lower with exercise preconditioning. At the same time, ejection fraction was significantly higher with exercise preconditioning, and NLRP3 was found to be negatively correlated with ejection fraction [82]. Aerobic exercise might reverse cardiac dysfunction partly through the P2X7R-inflammasome axis. HFD caused fibrosis and apoptosis in rat hearts, revealed by increased collagen deposition, disordered cells and the number of TUNEL-positive cells. At the same time, cardiac expressions of P2X7R, NLRP3, caspase-1 and serum IL-1β were also enhanced [58]. Twelve-week treadmill running effectively improved collagen deposition and cell disorder, and also inhibited the expressions of NLRP3, caspase-1, P2X7R and IL-1β in rat hearts [58].

## 4. Conclusions and Perspectives

It is now evident that the NLRP3 inflammasome is highly involved in the pathogenesis and progression of DCM. Targeting the NLRP3 inflammasome pathway in DCM is a potential therapeutic approach to manage the disease. However, the molecular mechanisms for activation of the NLRP3 inflammasome remain to be elucidated. As a recommended intervention method for DCM, exercise training has been proven to be effective in preventing cardiac inflammation, reversing cardiac structural changes and recovering cardiac function. However, the role that the NLRP3 inflammasome and P2X7R play in exercise-mediated alleviation of DCM remains to be explored. Further investigation in this area would help us better understand the underlying connection between exercise, inflammation, and DCM.

## Data Availability

Not applicable.
